# Dr. Jaeger's Clothing

**Published:** 1887-11-19

**Authors:** 


### DR. JAEGER'S CLOTHING.

We hope that, as Dr. Jaeger's representatives and the introducers of his Sanitary Woollen System into this country, you will allow us a short reply to your ably-written note in your issue of 12th instant. We thank you for your courteous commendation of the Jaeger system and garments, and we have only to offer explanation on two points. Dr. Jaeger did not originate a new kind of sheep or wool, but a new kind of fabric for underclothing, *i.e.*, the undyed stockinet, which has become so popular, and which is driving the old dyed flannel underclothing from the field. When we first introduced the Jaeger underclothing to this country, British manufacturers had not the requisite machinery wherewith to make it. But we never rested in our endeavours to procure the manufacture of our underclothing in this country, and we have now arrangements by which proper machinery and yarn are provided for making it, under strict control as to purity and other high qualities, in British factories by British workpeople. We trust that as you have written on the subject without knowing the last-mentioned fact, you will give equal publicity to this explanation of our position. Dr. Jaeger's Sanitary Woollen System Company (Limited).

Head Office, 95, Milton Street, E.C.

